# Human Identical Sequences, hyaluronan, and hymecromone ─ the new mechanism and management of COVID-19

**DOI:** 10.1186/s43556-022-00077-0

**Published:** 2022-05-20

**Authors:** Shuai Yang, Ying Tong, Lu Chen, Wenqiang Yu

**Affiliations:** 1 Laboratory of RNA Epigenetics, Institutes of Biomedical Sciences & Shanghai Public Health Clinical Center & Department of General Surgery, Huashan Hospital, Cancer Metastasis Institute, Shanghai Medical College, Fudan University, Shanghai, 200032 People’s Republic of China; 2 Shanghai Key Laboratory of Medical Epigenetics, Shanghai, 200032 People’s Republic of China

**Keywords:** Human Identical Sequences, Hyaluronan, Hymecromone, COVID-19, Ground-glass opacity

## Abstract

COVID-19 caused by SARS-CoV-2 has created formidable damage to public health and market economy. Currently, SARS-CoV-2 variants has exacerbated the transmission from person-to-person. Even after a great deal of investigation on COVID-19, SARS-CoV-2 is still rampaging globally, emphasizing the urgent need to reformulate effective prevention and treatment strategies. Here, we review the latest research progress of COVID-19 and provide distinct perspectives on the mechanism and management of COVID-19. Specially, we highlight the significance of Human Identical Sequences (HIS), hyaluronan, and hymecromone (“Three-H”) for the understanding and intervention of COVID-19. Firstly, HIS activate inflammation-related genes to influence COVID-19 progress through NamiRNA-Enhancer network. Accumulation of hyaluronan induced by HIS-mediated *HAS2* upregulation is a substantial basis for clinical manifestations of COVID-19, especially in lymphocytopenia and pulmonary ground-glass opacity. Secondly, detection of plasma hyaluronan can be effective for evaluating the progression and severity of COVID-19. Thirdly, spike glycoprotein of SARS-CoV-2 may bind to hyaluronan and further serve as an allergen to stimulate allergic reaction, causing sudden adverse effects after vaccination or the aggravation of COVID-19. Finally, antisense oligonucleotides of HIS or inhibitors of hyaluronan synthesis (hymecromone) or antiallergic agents could be promising therapeutic agents for COVID-19. Collectively, Three-H could hold the key to understand the pathogenic mechanism and create effective therapeutic strategies for COVID-19.

## Introduction

The ongoing pandemic of coronavirus disease 2019 (COVID-19) caused by SARS-CoV-2 infection has resulted in more than 6.2 million deaths globally according to the WHO Coronavirus Dashboard by April 27, 2022. Along with the increase in global infections and deaths, the economic burden and health threats caused by COVID-19 have been extremely acute [1–4]. In the world, there are different kinds of vaccines available for COVID-19 in various populations [5–9], including inactivated vaccines, live-attenuated vaccines, subunit vaccines, virus-like particles vaccines, viral vector-based vaccines, mRNA vaccines, and DNA vaccines. Especially, DNA vaccines are under development because of its long-term stability [10–12]. Recently, one DNA vaccine named INO-4800 against SARS-CoV-2 could stimulate durable immune responses in the phase 1 trial [13]. Unfortunately, the situation of COVID-19 pandemic is exacerbated by the emergence of SARS-CoV-2 variants. Since April 2021, the B.1.617.2 (Delta) variant, which has higher morbidity and transmissibility [14, 15]. Another B.1.1.529 (Omicron) variant was identified as the fifth variants of concern (VOC) by WHO on November 26, 2021 [16]. Currently, Omicron variant of SARS-CoV-2 has caused rapid epidemic expansion in many countries [17–20]. Specially, the mutational sites of receptor binding domain (RBD) regions in Omicron variant leads to its widespread escape from the responses of neutralizing antibodies [21–24]. Although vaccines theoretically prevent the transmission and infection of SARS-CoV-2 and are considered by the many to be the ultimate weapon against COVID-19 [14, 25–28], an increasing number of confirmed COVID-19 patients are alarmingly also vaccinated [15, 29–31]. Besides, some adverse reactions are reported in individuals vaccinated against COVID-19 [32], including myocarditis [33–36], thrombosis [37–41], adenopathy [42–44], abnormal cutaneous manifestations [45–47], and so on. Therefore, current circumstances indicate that there is still a long way to go to overcome COVID-19.

As the causative agent for COVID-19, SARS-CoV-2 is a new type of β-coronavirus (*β*-CoV) with a genome of about 30 kb and encodes at least 29 proteins [48–50]. The recent outbreaks of two viral pneumonia induced by *β*-CoVs infection are SARS-CoV in 2002 [51] and MERS-CoV in 2012 [52], respectively. Angiotensin-converting enzyme 2 (ACE2) is considered to be the common receptor for the cell entry of SARS-CoV and SARS-CoV-2 by binding to their surface spike (S) glycoprotein [53–55] while dipeptidyl peptidase 4 (DPP4) is the receptor for MERS-CoV-2 entry into cells [56–58]. In this case, DPP4 is considered as a potential receptor to binding the Spike protein of SARS-CoV-2 [59, 60]. Of note, a molecular docking study showed that the RBD of spike in SARS-CoV-2 had weakened interactions with DPP4 compared to MERS-CoV [61], indicating DPP4 was not a dominant receptor for SARS-CoV-2. Recently, numerous excellent reviews and comments related to COVID-19 have been published [62–69], including the methods for medical and laboratorial diagnosis [70–75], the transmission and epidemiology [76–79], the potential pathological mechanisms and clinical manifestations [80–86], the potential therapeutic strategies and management [87–93]. Excitingly, Dacheng Wei et al. developed a rapid and ultrasensitive method for the detection of SARS-CoV-2 [94], which just need 4 min and detected an ultralow concentration (one to ten copies in 100 μL biofluids). Notably, some experts thought that omicron variant of SARS-CoV-2 may overturn the COVID-19 pandemic based on its genetic mutation and clinical peculiarities [95–97]. Instead, the other thought that we need to face a grim reality for COVID-19 caused by omicron variant due to its rapid spread and evasion from the immune response [98–103]. Besides, most researches on the potential mechanisms underlying COVID-19 focus on the way for SARS-CoV-2 entry into host cells [104–107], the nonspecific and specific immune responses to SARS-CoV-2 infection [107–112], and the pathological consequences caused by SARS-CoV-2 infection [113–117]. However, there are still some important and fundamental scientific issues to be resolved. For instance, what are the vital pathogenic factors derived from SARS-CoV-2 for causing COVID-19? What is the material foundation of clinical manifestations of COVID-19?

It is well-known that *β*-CoVs replicate in double-membrane vesicles (DMVs) of cell cytoplasm [118–121]. Paulina Pawlica et al. found that SARS-CoV-2 could generate microRNA-like small RNA to silence host transcripts in cytoplasm and thus contribute to its pathogenesis [122]. However, recent researches indicated that SARS-CoV-2 RNA could be located in the host mitochondria and nucleolus [123, 124]. Moreover, numerous unknown transcripts of SARS-CoV-2 in infected Vero cells have been identified [125, 126]. Significantly, SARS-CoV-2 infection could cause human death even though it is not fatal to its potential hosts, including bats and pangolins [127, 128]. Frankly speaking, little is known regarding the function of SARS-CoV-2 RNA located in nucleus and the key factors involved in determining its pathogenicity in hosts. The recent research revealed that Human Identical Sequences of SARS-CoV-2 (HIS-SARS2) can promote COVID-19 progression by inducing hyaluronan accumulation through activating *HAS2* expression [129], which offers a novel insight into understanding the pathogenic mechanism of SARS-CoV-2.

To date, complete recovery from COVID-19 is still not optimistic despite the tremendous efforts that have been made. Based on previous literature and our understanding of COVID-19, this review discusses pertinent topics of public concern and provides an intensive exposition on SARS-CoV-2, especially with regards to the pathogenic mechanism and potential therapeutic strategies.

## The pathogenesis and intervention therapy of COVID-19 before Three-H  strategy

### The infection and transmission of SARS-CoV-2

The basic reproduction number R nought (*R0*), also called the basic reproductive ratio, refers to the average number of secondary infected individuals directly linked to the primary infected individual [130, 131]. Generally, *R0* is applied to evaluate the spread ability of communicable diseases, which indicates the intensity of infection and transmission for the infectious source (such as viruses and bacteria). Compared with the *R0* (2 to 5) of severe acute respiratory syndrome coronavirus (SARS) outbreak in 2003, the *R0* of COVID-19 caused by SARS-CoV-2 reached 1.5 to 6.49 [132, 133]. Remarkably, the mean R0 (5.08) of Delta variant is much higher than the R0 (2.79) of its ancestral strain [134], indicating the high communicability of COVID-19. Currently, the Omicron variant has rapidly became a dominating variant of SARS-CoV-2 instead of Delta variant [135]. Which factors then determine the infection and transmission of SARS-CoV-2?

**Fig. 1:**
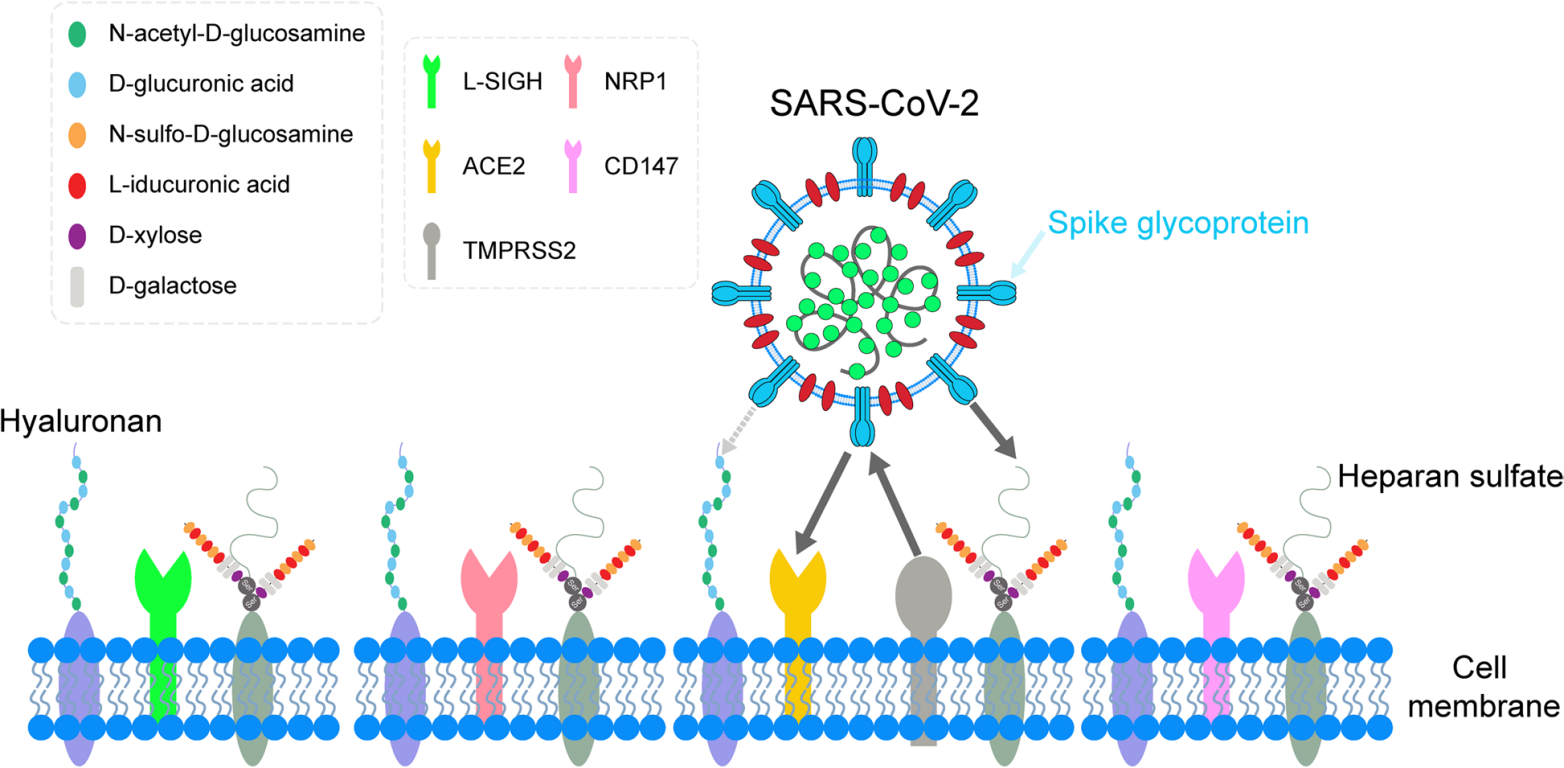
The model of SARS-CoV-2 entry into host cells. SARS-CoV-2 could initiate its attachment to the host cell membrane using the glycans (such as hyaluronan and heparan sulfate). Then, the interaction between spike glycoprotein and diverse receptors gives the susceptibility SARS-CoV-2 needs to invade host cells. Notably, hyaluronan is universal in the extracellular matrix of different cells, which may explain the infection of SARS-CoV-2 into cells only with a high viral load

The cell entry of SARS-CoV-2 is reported to be dependent on the ACE2 receptor with the help of TMPRSS2 [136–138]. Especially, the affinity between the S protein of SARS-CoV-2 and ACE2 receptor is 10–20 times that of SARS-CoV-1 [139–141], which gives the susceptibility SARS-CoV-2 needs to invade cells. Surprisingly, the expression of ACE2 is upregulated in the lungs of patients with severe COVID-19 [142–144]. Consistent with this finding, HIS of SARS-CoV-2 can stimulate ACE2 expression [129], implying that SARS-CoV-2 can promote cell entry through upregulating receptor expression by itself. As one of the glycans, heparan sulfate can interact with the RBD of the SARS-CoV-2 S glycoprotein to facilitate the binding of S protein to ACE2 [145]. Consist with this result, the infection of SARS-CoV-2 pseudovirus is significantly decreased in 293 T-hACE2 cells after treatment with heparin [146]. Noteworthily, SARS-CoV-1 and MERS-CoV can also utilize glycans mediated by their initial attachment to the host cell membranes [147, 148], indicating the importance of glycans in virus infection. Interestingly, endothelial cells can be infected by SARS-CoV-2 only with a high viral load [149]. Moreover, the diversity of receptors facilitates their facile invasion into cells (Fig. 1), such as ACE2 [136], NRP1 [150], CD147 [151], and L-SIGN [152]. As one of the RNA viruses, the emergence of variants also enhances their high infectivity. For example, the widespread transmission of G614 variants in Europe and America is 3–9 times higher than that of the original D614 strain [153].

### The re-positive appearance of SARS-CoV-2

The re-positive cases of COVID-19 are usually defined as confirmed cases with SARS-CoV-2 infection after discharge. Different cohort studies have shown that the re-positive ratio of SARS-CoV-2 ranges from 2.4% to 69.2% [154–158]. Notably, most re-positive cases are asymptomatic or mild after discharge [154, 155, 159]. Moreover, the re-positive cases tend to be younger populations, given its median average age at 28 years old [156]. Additionally, the viral load of SARS-CoV-2 was very low in the re-positive cases, as is their transmission risk [158, 159]. From these characteristics of re-positive cases, we speculated that a small amount of SARS-CoV-2 is still residual in the epithelial cells of the nasopharynx even if the clinical symptoms of COVID-19 have receded after treatment. In other words, the patients of re-positive cases had always carried SARS-CoV-2 rather than being truly “re-positive.” This gap in knowledge can be caused by the limited sensitivity of current detection methods for SARS-CoV-2.

Notably, the re-positive appearance of SARS-CoV-2 may hint at the “friendship” between SARS-CoV-2 and the epithelial cells of the nasopharynx. It is well-known that influenza viruses can disrupt the mucosal barrier resulting in coinfection with common strains of bacteria [160–162], indicating that influenza viruses may cause damage to epithelial cells in the respiratory tract. However, the rate of COVID-19 cases with coinfection of bacteria is considerably low [163–166], further supporting the “friendship” between SARS-CoV-2 and the epithelial cells of the nasopharynx. Accordingly, one of the most common symptoms of COVID-19 is dry cough without sputum [167–169], which has been used to accurately diagnose COVID-19 via artificial intelligence [48].

### The cytokine storm in COVID-19

The hyperactive immune responses in COVID-19 patients promote the release of a large number of pro-inflammatory cytokines and further stimulate excessive inflammatory reaction, also known as the cytokine storm [170], which induces acute respiratory distress syndrome (ARDS). It is reported that high concentrations of cytokines(such as IL-1β, TNFα, IL-6, and IL-8) and chemokines (such as CCL2, CCL-5, IFNγ-induced protein 10 (IP-10), and CCL3) are detected in plasma and BALF and are associated with the occurrence and poor clinical outcomes of ARDS, such as mortality rate [171–176]. Apoptosis and other type of cell deaths could be another factors to cause ARDS, which has been reviewed in detail elsewhere [177]. For example, apoptosis mediated by the activation of Fas/Fas ligand pathway contributes to ARDS [178–180]. Currently, many systematic reviews have concentrated on cytokine storm in COVID-19 and discussed its potential mechanisms including signaling pathways [181–187], which mainly emphasized the important roles of immune cells in cytokine storm. However, it isn’t still clearly elucidated about the pathogenesis of cytokine storm. In the following section, we will discuss the distinct perspectives on the underlying mechanisms for cytokine storm in COVID-19 based on current clinical observations and experimental findings.

### Spike protein of SARS-CoV-2 may stimulate allergic reaction during COVID-19

The aberrant cutaneous manifestations in COVID-19 has been found in a multitude of retrospective studies [188–190], such as the erythematous rash, urticaria and maculopapular eruptions, which are also the typical symptoms of allergic reactions. It is well-known that abnormal levels of IgE and histamine are universal indicators to assess allergic reactions [191–193]. In a retrospective study on COVID-19 [194], the level of IgE is significantly increased in non-survivors (71.30 IU/mL), compared to survivors (42.25 IU/mL). Similarly, 119 of 303 (39%) COVID-19 patients with elevated serum IgE [195]. Particularly, the dynamic change of IgE is closely similar to IgM against SARS-CoV-2 [196]. Some excellent reviews have recently highlighted the importance of histamine and its receptors in COVID-19 [197, 198]. Particularly, SARS-CoV-2 could activate mast cells to secrete histamine [199]. At present, there are four known receptors of histamine, designated as H1/H2/H3/H4 receptors (H1R/H2R/H3R/H4R). Although H1 and H2 receptors are relevant to allergic inflammation and gastric acid secretion, respectively [200], both antagonists of H1R and H2R are therapeutic agents for acute allergic reactions in a clinical set [201]. Excitingly, the antagonists of H1/H2 receptor have been proven to improve outcomes of COVID-19 [198, 202]. Above all, allergic responses could appear in some COVID-19 patients, which were potentially elicited by SARS-CoV-2 infection. In this case, what are the potential allergens in COVID-19 after SARS-CoV-2 infection?

Here, the S protein of SARS-CoV-2 may have a potential role as an allergen by analyzing the phenotype and potential molecular mechanisms after vaccination. Since the beginning of COVID-19 outbreak, the spike protein of SARS-CoV-2 has been considered as a foremost target for COVID-19 vaccine development. For example, one of the nCoV-19 vaccines, ChAdOx1, is a full-length virus vector of S-protein [203]. The occurrence of thrombocytopenia and thrombosis in individuals after ChAdOx1 vaccination has attracted public attention [204, 205]. In particular, survivors are discharged from the hospital on day 12 after prednisolone treatment [205], a typical drug used for allergic diseases. Besides, anti-spike binding was detectable in all these individuals while the levels of antibodies against the S protein are varied [205], suggesting the S protein was produced alongside the emergence of the syndrome. Another nCoV-19 vaccine, NVX-CoV2373 is a recombinant nanoparticle vaccine containing the trimeric full-length spike glycoproteins of SARS-CoV-2 (rSARS-CoV-2) and Matrix-M1 adjuvant [206]. During the phase 1–2 trial of NVX-CoV2373, only vaccination of 25 μg rSARS-CoV-2 can induce some mild symptoms after the second dose of vaccination including erythema, redness, induration, or swelling [206], which provides direct evidence for the S protein as an allergen. In line with these findings, the safety assessment on the COVID-19 mRNA vaccine showed that the suspected adverse reactions primarily occurred after the second dose of COVID-19 vaccination [207]. Surprisingly, the spike protein of SARS-CoV-2 could directly induce the release of proinflammatory cytokines (such as TNFα) and apoptosis in THP-1-like macrophages [208], indicating that S protein may serve as a pathogenic substance. Moreover, the S protein accelerated the expression of pro-thrombotic molecules in pulmonary endothelial cells [209], further hinting the pathogenicity of S protein. Notably, a recent work found that high-sulfated hyaluronan could inactivate SARS-CoV-2 including Alpha and Beta variants by stable binding [210], emphasizing the interaction between hyaluronan and SARS-CoV-2. The structure of monomers is similar between hyaluronan and heparin. Given that heparan sulfate can steadily bind to the RBD of S proteins [145], there could be a similar interaction between hyaluronan and the S protein. Alarmingly, some individuals with hyaluronan dermal fillers had hypersensitivity reactions after SARS-CoV-2 infection or COVID-19 vaccination [211–217], indicating the combination of hyaluronan and S protein may trigger the allergic reactions. Besides, COVID-19 vaccination led a distinct hepatitis mediated by CD8 T cell-dominant immune [218], which may attribute to the long-term expression of S protein from the mRNA vaccination. Interestingly, the binding of hyaluronan to CD44 could regulate the CD8 T cell response [219], further suggesting that the complex of hyaluronan and S protein may induce the hepatitis in individuals after COVID-19 vaccination. Collectively, all these signs suggest that the S protein of SARS-CoV-2 could serve as allergen and cause the allergic response in certain patients, further aggravating their COVID-19 symptoms or inducing adverse reactions toward vaccination.

### Current therapeutic strategies for COVID-19

At present, symptomatic supportive treatment is still the primary therapeutic strategy for COVID-19 clinically [62]. Many potential therapeutic strategies have been proposed to combat COVID-19 [220–229], such as antiviral treatment, immunological therapy, Chinese medicinal therapy, and anti-inflammatory therapy. Interestingly, electric stimulation may be a subsidiary approach to improve COVID-19 outcomes by increasing the penetration of antiviral drugs [230]. Recently, these therapeutics for COVID-19 have been well-summarized in an updated review [231]. Given the emergency circumstances of COVID-19, some drugs have been urgently approved for COVID-19 treatment (Table 1), which still need further clinical trials to confirm their effectiveness. The development of vaccines against SARS-CoV-2 is considered a key approach to fighting the COVID-19 outbreak. As of April 23, 2022, 153 COVID-19 vaccine candidates are in human clinical trials while 197 COVID-19 vaccine candidates are in preclinical development [232]. Numerous clinical trials of potential therapeutic drugs on COVID-19 are ongoing in the world. Yet unfortunately, there are still few drugs available for COVID-19 treatment in clinical practice other than dexamethasone, which promotes us to further investigate the pathogenic mechanism of COVID-19 and develop new therapeutic strategies.

**Table 1:** The urgent approved drugs for COVID-19 treatment

Schemes	Classes	Dosage forms	Clinical trial numbers	References
Remdesivir	Antiviral drug	I.V.	NCT04292899 (Phase 3) NCT04292730 (Phase 3) NCT04401579 (Phase 3) NCT04280705 (Phase 3)	[233–236]
Baricitinid plus Remdesevir	Antiviral drug	I.V./Oral	NCT04970719 (Phase 3) NCT04401579 (Phase 3) NCT04640168 (Phase 3)	[237]
Paxlovid	Antiviral drug	Oral	NCT04960202 (Phase 3) NCT05011513 (Phase2/3) NCT05047601 (Phase 3)	[237–239]
Molnupiravir	Antiviral drug	Oral	NCT04575584 (Phase 2/3) NCT04575597 (Phase 2/3) NCT04939428 (Phase 3) NCT04405570 (Phase 2) NCT05195060 (Phase 3)	[240–242]
BRII-196/BRII-198	Monoclonal antibodies	I.V.	NCT04787211 (Phase 2) NCT04518410 (Phase 2/3) NCT04501978 (Phase 3)	[237, 243]
Bebtelovimab	Monoclonal antibodies	I.V.	NCT04634409 (Phase 2)	[237, 238]
Bamlanivimad plus Etesevimab	Monoclonal antibodies	I.V.	NCT05205759 (Phase 3) NCT04790786 (Phase 3) NCT04634409 (Phase 2) NCT04427501(Phase 2)	[237, 238, 244–246]
Casirivimab plus imdevimab	Monoclonal antibodies	I.V.	NCT05205759 (Phase 3) NCT05074433 (Phase 3) NCT04425629 (Phase 3) NCT04790786 (Phase 3) NCT04452318 (Phase 3) NCT04518410 (Phase 2/3)	[244, 247–251]
Sotrovimab	Monoclonal antibodies	I.V.	NCT04913675 (Phase 3) NCT04779879 (Phase 2) NCT04790786 (Phase 3) NCT04381936 (Phase 2/3)	[237, 244]
Convalescent plasma	Plasma	I.V.	NCT04747158 (Phase 2/3) NCT04649879 (Phase 2/3) NCT04433910 (Phase 2) NCT04355767 (Phase 3) NCT04547660 (Phase 3) NCT04345523 (Phase 2) NCT04359810 (Phase 2) NCT04381858 (Phase 3) NCT04747158 (Phase 2/3) NCT04425915 (Phase 3) NCT04362176 (Phase 3) NCT04332835 (Phase 2/3)	[252–261]
Evusheld	Monoclonal antibodies	I.M.	NCT04625725(phase 3) NCT04625972(phase 3)	[262]
VV116	Antiviral drug	Oral	NCT05242042(phase2/3) NCT05341609(phase3)	[263, 264]

## HIS as a nuclear acid factor from SARS-CoV-2 for the progression of COVID-19

It is well known that protein encoded from the virus genome is the main driving factor in virus infection and disease development. Since the virus also contain nuclear acid, it would be interesting to investigate whether nuclear acid, especially in its non-coding regions, can also act as a pathogenic factor in virus infection, especially for SARS-CoV-2.

### HIS-SARS2 could be a crucial pathogenic factor of SARS-CoV-2

**Fig. 2:**
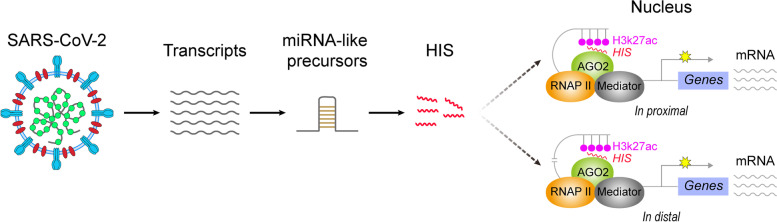
HIS-SARS2 activates inflammation-related genes by targeting enhancer. After infection, SARS-CoV-2 can generate plenty of unknown transcripts, which may serve as the miRNA-like precursors and produce HIS. With the help of AGO2, HIS could bind to their targeting enhancers in the human genome. Subsequently, the interaction between enhancer and RNA polymerase II (RNAP II) mediated-mediators will promote the transcription of inflammation-related genes in proximal or in distal

Human Identical Sequences of SARS-CoV-2 (HIS-SARS2) are greater than 20 bp and are entirely identical sequences between SARS-CoV-2 and human genomes [129]. They function as miRNA-like RNA. Intriguingly, it has been reported that multiple viruses could produce miRNA-like non-coding RNAs during their infection [265–269]. For instance, the small viral RNAs (svRNAs) encoded by SARS-CoV could repress host mRNA expression by targeting 3′-UTR of specific transcripts [270]. Specially, SARS-CoV-2 can generate viral miRNAs in relation to the cellular metabolism and biosynthesis in host cells [271]. Recently, a noncoding RNA produced from the ORF7a in SARS-CoV-2 genome was demonstrated to decrease the host transcripts (such as BATF2) via target slicing [122]. Inversely, HIS-SARS2 could activate gene expression by targeting enhancer in human [129], which significantly overlap with the aberrant expressed genes found in bronchoalveolar lavage fluid (BALF) of COVID-19 patients [272]. Particularly, HIS-SARS2 could upregulate inflammation-associated genes in transformed human embryonic kidney cell HEK293T, human fetal lung fibroblast cell MRC5, and human umbilical vein endothelial cell (HUVEC), which is consistent with the finding that the infection of SARS-CoV-2 could lead to multiple organ damage (such as lung, kidney, and liver) by stimulating an inflammation response [273–276]. Moreover, HIS-SARS2 destroyed the function of mitochondria by increasing *CYB5A* and *TIMM21*, which may cause the mitochondria dysfunction related to COVID-19 pathogenesis [277–280]. Moreover, the major enzyme for hyaluronan synthesis, *HAS2*, was activated by HIS-SARS2, which promoted hyaluronan accumulation in severe COVID-19 patients [129, 281, 282]. Notably, SARS-CoV-2 RNA was detected in the plasma of COVID-19 patients using droplet-based digital PCR [283], raising a possibility that HIS-SARS2 could be transported into the distal cells and via hematologic system. Therefore, HIS-SARS2 exert an important role in the pathogenicity of SARS-CoV-2 during infection (Fig. 2).

Strikingly, SARS-CoV-2 infection does not affect the health of bats and pangolins [127, 128]. Some identical sequences were also found between SARS-CoV-2 and its potential hosts’ genomes, which were termed as “Host Identical Sequences (HIS)”, while no identical sequences were identified between the genome of SARS-CoV-2 and chicken. This indicates that HIS from SARS-CoV-2 may be helpful when tracing to its mediated hosts.

Most importantly, we put forward the hypothesis of nucleic acid pathogenicity based on the discovery of HIS, which is that the identical sequences between the genome of viruses and hosts (such as human) probably hold the key for viruses to infect hosts and cause diseases. In fact, there are also identical sequences between the genome of human and other pathogenic viruses, including HIV, Ebolavirus, and Zika virus [129], which provide additional support for our hypothesis. The HIS in different viruses deserve further efforts toward clarifying their potential functions, such as acting as important therapeutic targets for these associated diseases.

### Cytokine storm may be triggered by SARS-CoV-2 rather than the passive response of host

As we all know, the immune responses divide into innate and adaptive responses, which are indisputably activated by SARS-CoV-2 infection in COVID-19. Particularly, the adaptive immune response is believed to be the most potent approach to clearing SARS-CoV-2 [110, 284–286], which is contradictory to a recent report that no IgG antibody against SARS-CoV-2 is detected in 18% of COVID-19 patients even though the average testing time between IgG positive and negative groups is close [287]. Moreover, COVID-19 patients with the second infection have more severe clinical presentation than their first infection [288–290], indicating that adaptive immune response may not exert a dominant role in combating SARS-CoV-2. In fact, many individuals with SARS-CoV-2 infection are capable of clearing the virus under asymptomatic situations [291–293], which implies that innate immunity may hold the key to defeating SARS-CoV-2 as the first defense line against environmental pathogenic substances. In addition, different single-cell omics analyses of COVID-19 patients suggest that the cellular components of innate immune (such as macrophages and monocytes) could determine COVID-19 severity [294, 295]. Thus, innate immunity may play a dominant role in responding to SARS-CoV-2 infection rather than adaptive immunity during the COVID-19 process, which could be harnessed to mitigate COVID-19.

**Fig. 3:**
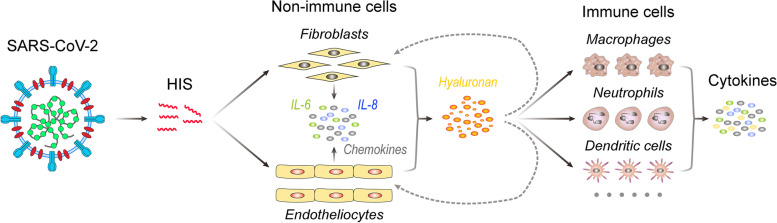
The formation of cytokine storm in severe COVID-19. In fibroblasts and endotheliocytes, HIS generated by SARS-CoV-2 could activate HAS2 expression and promote the hyaluronan. Then, hyaluronan is degraded into different fragments. The low molecular weight of hyaluronan can bind to its receptors in non-immune cells and immune cells and produce different cytokines, including IL-6, IL-8, and chemokines, resulting in cytokine storm in COVID-19

Currently, it is a common consensus that various cytokines in COVID-19 are generated by diverse immune cells involved in innate immunity (such as neutrophils and macrophages) and adaptive immunity (such as adaptive B and T lymphocytes) [296]. There are many significant increases of cytokines in severe COVID-19 patients [296–298], including TNF-α, IL-6, IL-8, and IL-10. Honestly, we have unwittingly ignored that non-immune cells (such as endothelial cells and fibroblasts) can also produce cytokines [299, 300], which may contribute to the cytokine storm in COVID-19. For example, pulmonary endothelial cells could produce IL-6 by sensing SARS-CoV-2 infection in the adjacent epithelium [149]. Similarly, HIS-SARS2 could activate inflammation-associated genes in human umbilical vein endothelial cells [129]. In line with these findings, circulating endothelial cells were significantly more relevant to IL-6 in severe COVID-19 patients [301]. Notably, fibroblasts could also produce pro-inflammatory cytokines and participate in the persistence of inflammation [302], which may underly the multi-organ fibrosis of COVID-19 patients [303]. Especially, HIS-SARS2 could upregulate HAS2 in human fetal lung fibroblast cells and promote the synthesis of hyaluronan [129], a crucial mediator for inflammation, which may be connected to the pulmonary fibrosis of COVID-19 [304]. Notably, the binding of hyaluronan to CD44 can induce the production of IL-6 and IL-8 in human dermal fibroblasts [305]. In addition, the interaction between hyaluronan and its receptors facilitates cytokine production (such as IL-6 and IL-8) in immune cells [306–308], including macrophages, neutrophils, and dendritic cells. Together, these findings hint that cytokines released by non-immune cells may trigger cytokine storm in severe COVID-19 (Fig. 3). Given that fibroblasts are widely distributed in various tissues and organs, we believe that fibroblasts may be a major source of cytokine in COVID-19.

## Hyaluronan functions as an essential inducer for the development and severity of COVID-19

### Hyaluronan could be a main contributor underlying the clinical manifestations of COVID-19

COVID-19 has various clinical symptoms. The most common symptoms of COVID-19 are fever, dry cough, and shortness of breath [167]. Based on chest CT, ground-glass opacity (GGO) or consolidations exist in the lungs of COVID-19 patients [309]. Lymphopenia and elevated C-reaction protein (CRP) are two of the most common laboratory abnormalities in hospitalized COVID-19 patients [310]. Meanwhile, severe COVID-19 patients develop ARDS [311]. Notably, COVID-19 can impair the function of multiple organs (such as heart, brain, lung, liver, and kidney) and the coagulation system [62]. Additionally, COVID-19 patients show some neurological complications and symptoms (such as headache, encephalitis, and intracerebral hemorrhage) [312]. However, the foundation underlying the clinical manifestations of COVID-19 is still ambiguous.

**Fig. 4:**
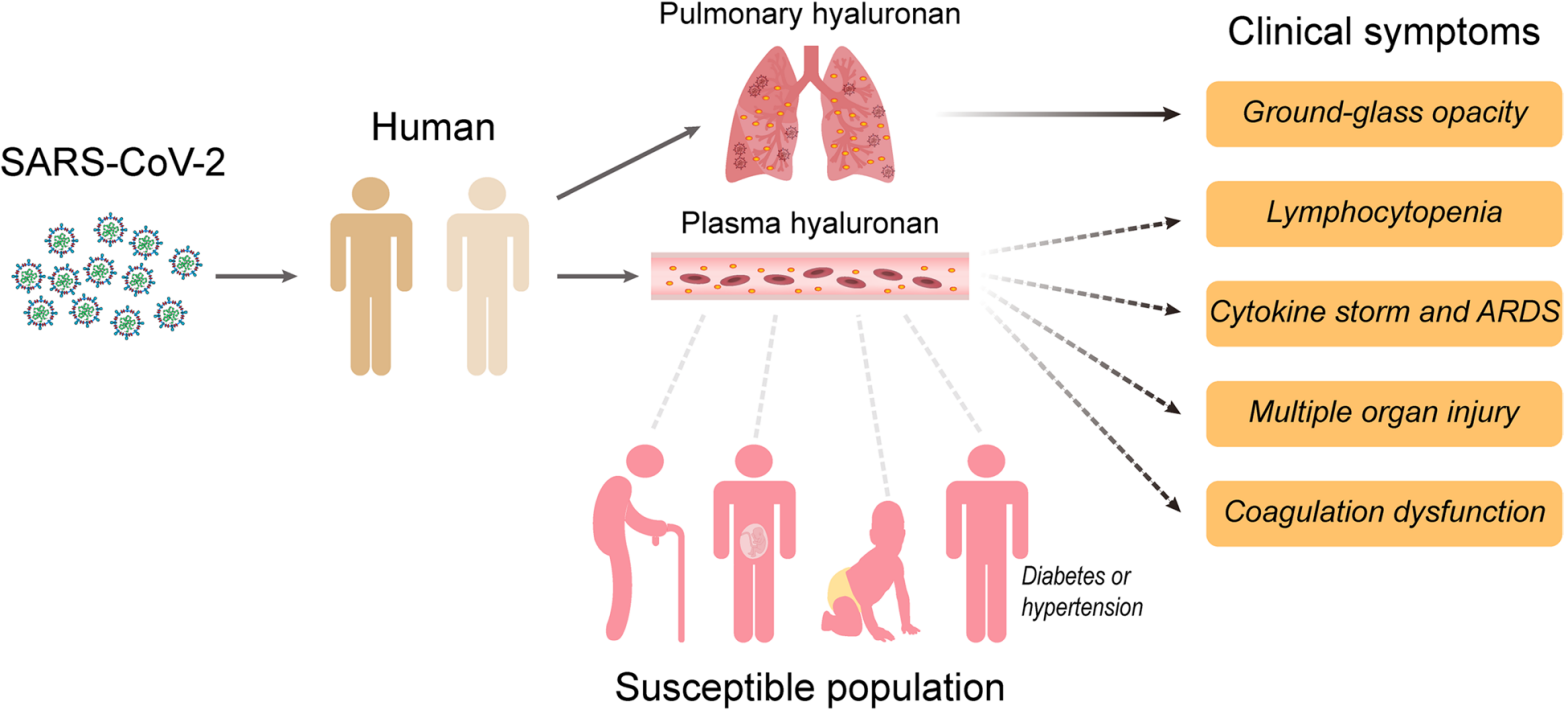
The potential relationships between hyaluronan and COVID-19. The infection of SARS-CoV-2 accelerates the production of hyaluronan in blood and lung in COVID-19 patients. The accumulation of hyaluronan in lung directly causes the ground-glass opacity, which further develops consolidation. Additionally, the plasma hyaluronan is closely related to other clinical symptoms including lymphocytopenia, cytokine storm, ARDS, multiple organ injury, and coagulation dysfunction. Notably, the most susceptible population of SARS-CoV-2 have a high level of plasma hyaluronan

Recently, a few studies on metabolic profiles have revealed that the metabolism of carbohydrates, fats, and proteins are dysregulated in COVID-19 patients [313–317], which may provide us some important indication. Specially, gene alterations involved in the metabolism of hyaluronan, glycosaminoglycan, and mucopolysaccharides are excessive in SARS-CoV-2 infected bronchoalveolar cells [317]. In line with this finding, there is a significant increase of hyaluronan in patients with severe and critical COVID-19 [129, 281], indicating that hyaluronan is related to the COVID-19 clinical process. According to the recent results combined with previous literature, hyaluronan may be crucial to the material foundation of COVID-19 clinical symptoms (Fig. 4).

#### Hyaluronan may be an important substance for ground-glass opacity in lung

GGO is a typical presentation of COVID-19 patients based on chest CT, which can further develop into consolidations. Autopsy from three patients with COVID-19 showed that hyaluronan is accumulated in the lung alveoli [318]. Similarly, hyaluronan is also abundant in the respiratory secretions of COVID-19 patients [319]. Strikingly, hyaluronan positively relates to the volume of extravascular water in normal animal lungs [320], which may be due to its ability to absorb a large volume of water [321]. This water absorption characteristic of hyaluronan may be a cause for the jelly-like substance formation present in the lungs of severe COVID-19 patients [322]. Moreover, intratracheal instillation of hyaluronan directly causes pulmonary ground-glass opacity (GGO) or consolidations in mice [323]. Therefore, these evidences suggest that the GGO or consolidations in COVID-19 patients’ lungs contribute to the increase in hyaluronan.

#### Hyaluronan may cause the lymphocytopenia in patients with COVID-19

Lymphocytopenia is a syndrome defined as the loss of lymphocyte in peripheral blood. The reduction of lymphocytes is revealed to be lower in COVID-19 patients with higher levels of hyaluronan [129]. The increased hyaluronan is positively related to the elevated CRP, but negatively related to decreased lymphocytes in COVID-19 patients, which may be due to the reduction in total T lymphocytes [324]. Intriguingly, the interaction between hyaluronan and its ligand CD44 can cause T cells death when they are activated [325]. In fact, the infection of SARS-CoV-2 can rapidly activate CD4+ T lymphocytes [326]. Thus, the reduction of T lymphocytes mediated by hyaluronan may underlie the foundation of lymphocytopenia in patients with COVID-19.

#### Hyaluronan may promote acute respiratory distress syndrome in COVID-19

ARDS is a clinical syndrome characterized by hypoxemia and nonhydrostatic pulmonary edema [327], one of the leading causes of COVID-19 death. Generally, ARDS is thought to be the impairment of pulmonary vascular permeability, which increases lung mass as caused by acute diffuse inflammatory lung injury. Plenty of studies have revealed that hyaluronan can serve as an essential mediator for inflammation and vascular homeostasis [328, 329], which may underlie the inflammatory and vascular permeability impairment in COVID-19 patients with ARDS. These insights are supported by the emergence of hyaluronan in the lung alveoli of patients with ARDS [318]. Especially, HIS-SARS2 can upregulate the expression of a key hyaluronan synthase *HAS2* in MRC5 and HUVEC [129], suggesting that the SARS-CoV-2 infection could directly induce hyaluronan accumulation in lungs. Consistent with these results, pulmonary microvascular endothelial cells could produce hyaluronan to directly destroy the endothelial barrier when they are exposed to the COVID-19 environment [330]. The binding of hyaluronan may mediate the disruption of endothelial cell barrier to its receptor HABP2 [331]. Hence, beyond triggering the pulmonary inflammatory, dysfunction of the pulmonary endothelial barrier induced by hyaluronan could account for ARDS.

#### Hyaluronan may trigger multiple organ injury and coagulation system dysfunction

SARS-CoV-2 has been demonstrated to infect diverse cell types in different tissues [150, 332–334], including lungs, kidneys, brain, and heart. Interestingly, hyaluronan is widely distributed in all parts of the body and exercises a myriad of biological functions in different cell types [335]. Particularly, HIS-SARS2 can induce hyaluronan production via stimulating HAS2 expression in diverse cells associated with lung, blood vessel, and kidney [17], implying the inflammation trigged by hyaluronan may be the cause of multiple organ injury. In addition, 71% of 183 COVID-19 patients who passed away had diffuse intravascular coagulation [336]. Notably, HABP2, a receptor of hyaluronan is involved in blood coagulation [337], which may also play a key role in the dysfunction of the coagulation system in response to the increased hyaluronan level in COVID-19 patients.

### Hyaluronan is a pivotal connection between COVID-19 and its risk factors

The case fatality rate (CFR) of COVID-19 worldwide in 219 countries is approximately 2.1% based on the WHO Coronavirus Dashboard by 22 August 2021. There are many risk factors (such as advanced age, diabetes, hypertension, and cancer) for COVID-19 mortality [338]. Specially, pregnant women and newborns are susceptible to SARS-CoV-2 and can develop severe COVID-19 [339, 340]. Additionally, there are some long-term sequelae of COVID-19 [341, 342], such as severe fatigue, loss of sense of smell or taste, and skin rash. Of note, female sex and older age are related to the risk of persistent symptoms in long COVID [343]. However, the intrinsic relationship between COVID-19 and its risk factors are still unclear.

Remarkably, hyaluronan could help explain the connection between COVID-19 and some of its risk factors. It is reported that serum hyaluronan level of newborns (0–7 day) and elderly people (>60 years) are higher than the other ages in 585 healthy individuals [344]. The higher level of hyaluronan could be associated with the risk of long-term sequelae of COVID-19 in older people. Similarly, the serum hyaluronan level in diabetic patients (83.6 ± 5.6 ng/mL) was significantly higher than in normal subjects (41.7 ± 12 ng/mL) [345]. In addition, the elevation of plasma hyaluronan was found in patients with pulmonary hypertension and cancer [346, 347]. Moreover, there is a gradual increase of serum hyaluronan level in women during pregnancy [348], which may partly explain the higher probability of COVID-19 sequelae in female. And lastly, individuals who have received vaccination but died from COVID-19 did so from major cerebral hemorrhage [204]. All these individuals have a high level of platelet factor 4 (PF4). Notably, PF4 can stimulate the release of histamine from basophils and mast cells [349, 350], which could further accelerate the hyaluronan synthesis [351]. Surprisingly, hyaluronan is capable of regulating vascular integrity [352], which may caused the fatal intracranial hemorrhage in vaccinated individuals. Recently, it is reported that the hepatitis of unknown aetiology occurred in five young children infected with SARS-CoV-2 in Scotland [353], which may be attributable to the accumulation of hyaluronan in liver [354]. Thus, we can reasonably infer that a high level of hyaluronan in these individuals may provoke a more violent response after SARS-CoV-2 infection, further exacerbating COVID-19 symptoms (Fig. 4).

### Hyaluronan could serve as an important indicator for COVID-19

Serum hyaluronan is usually a non-invasive test to diagnose liver cirrhosis [355], which means that it is exceptionally convenient to detect hyaluronan in clinical. Combined with the potential roles of hyaluronan in COVID-19, hyaluronan may become a useful clinical indicator for COVID-19. For one, hyaluronan can predict the progression of COVID-19, which is helpful for physicians to determine which patients would require special attention. Secondly, hyaluronan can act as a biomarker to appraise the prognosis of COVID-19. And lastly, hyaluronan can initially screen individuals vaccinated against COVID-19, which may reduce adverse reactions in certain individuals.

## Hymecromone as a prospective therapeutic agent for overcome with COVID-19

**Fig. 5:**
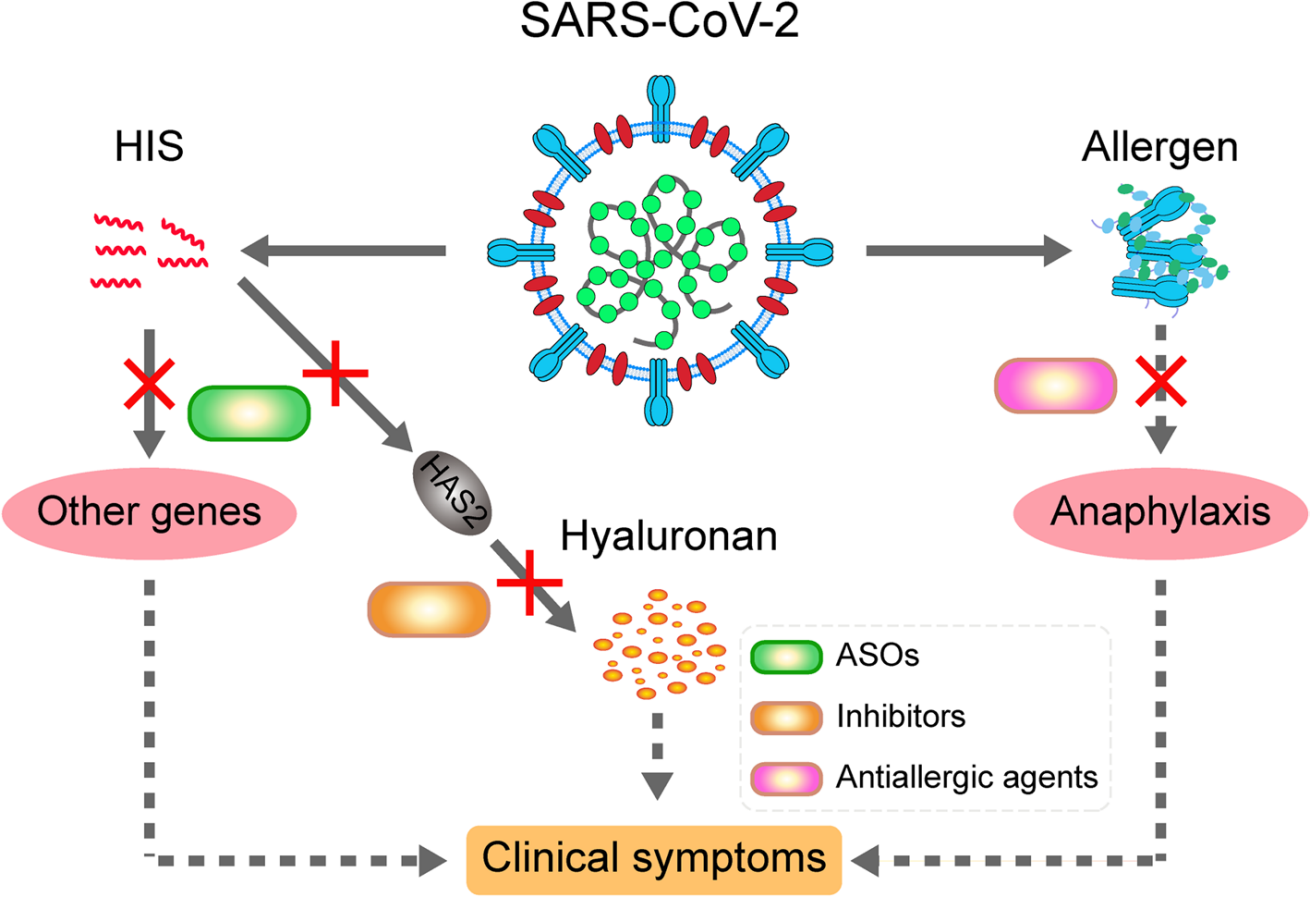
The potential strategies for COVID-19 therapy. There are two potential approaches to cause the clinical symptoms of COVID-19. In one hand, the generation of HIS from SARS-CoV-2 could activate inflammation-related genes (such as *HAS2*) and induce the production of hyaluronan. On the other hand, the spike protein of SARS-CoV-2 could trigger the allergic reaction in some individuals with COVID-19. Accordingly, we could use antisense oligonucleotides (ASOs) of HIS-SARS2, inhibitors of hyaluronan synthesis, and antiallergic agents to block these approaches and thereby facilitate the recovery of clinical symptoms in COVID-19

Here, we proposed three promising therapeutic agents for COVID-19 treatment on the basis of our recent studies and novel insights into COVID-19, including antisense oligonucleotides (ASOs) of HIS-SARS2, inhibitors of hyaluronan synthesis, and antiallergic agents (Fig. 5).

### ASOs of HIS-SARS2

ASOs are defined as synthesized oligonucleotides measuring 12–30 nucleotides, which are designed to bind to RNA based on base pairing rules [356]. Along with the improvement in technology, ASOs have been used as a therapeutic drug since the late 1980s. To date, several ASO drugs have been approved by the Food and Drug Administration (FDA) to treat different diseases [357]. For example, a 30-mer morpholino ASO, eteplirsen obtained the provisional approval by FDA to treat Duchenne Muscular Dystrophy [358]. Notably, antisense morpholino oligomers targeting the transcription-regulatory sequence (TRS) regions of SARS-CoV could inhibit the production of SARS-CoV [359]. Similarly, the antisense peptide nucleic acid targeting the highly conserved PRF signal of SARS-CoV could significantly suppress its replication [360]. Recently, the importance of ASOs in combating COVID-19 have been recognized due to their high target specificity and rapid development [361]. Especially, the appearance of nanotechnology could facilitate the delivery of ASOs to their target sites [362]. However, it is crucial to identify the potential targets of SARS-CoV-2.

The recent research has clarified the key roles of HIS-SARS2 in response to SARS-CoV-2 infection. In particular HIS-SARS2 upregulated genes associated with inflammation whereas their antagomirs abolished their activation [129], suggesting the blocking of HIS-SARS2 could be conducive to alleviating the inflammatory response in COVID-19. Therefore, HIS-SARS2 are candidate targets when designing ASOs, which hold great potential in treating COVID-19.

### Inhibitors of hyaluronan synthesis

The concentration of hyaluronan is significantly higher in patients with severe COVID-19 [129, 281]. As mentioned above, elevated hyaluronan in COVID-19 could cause most clinical manifestations (such as GGO, lymphopenia, and ARDS) and establish a subtle connection with the risk factors, indicating that hyaluronan could be a key therapeutic target for COVID-19. In fact, this insight is also supported by certain medications for COVID-19 that have already been proven effective in clinical trials. Dexamethasone and Metformin are reported to significantly reduce the CFR of severe COVID-19 patients [363–365], which may be partly attributed to their effects in rapid decrease of hyaluronan [366, 367]. Consequently, inhibitors of hyaluronan synthesis are promising therapeutic agents for COVID-19.

4-methylumbelliferone (4-MU) is a coumarin derivative that can suppress the hyaluronan synthesis by downregulating the mRNA levels of hyaluronan synthases and depleting their substrate UDP-glucuronic acid [368]. Fortunately, there is an approved prescription drug of 4-MU, called hymecromone, which is used for biliary spasm treatment [369]. In a small sample clinical trial, hymecromone has been verified to accelerate the recovery of COVID-19 patients via the promotion of lymphocyte recovery and pulmonary lesion absorption [323]. In other words, hymecromone could be an efficient clinical prescription to block COVID-19 progression.

### Antiallergic agents

The elevation of serum IgE in COVID-19 patients indicates SARS-CoV-2 infection can stimulate an allergic reaction in some individuals. We proposed above that the S protein of SARS-CoV-2 may serve as an allergen to stimulate an allergic reaction. As such, antiallergic agents could be potential candidates for COVID-19. In general, common clinical antiallergics are antihistamines (such as Diphenhydramine, Promethazine, and Chlorpheniramine) and corticosteroids (such as Dexamethasone) [370, 371]. In addition, the monoclonal antibodies of anti-IgE are alternative agents for anaphylaxis [372]. These antiallergic agents may be new weapons against COVID-19.

## Concluding remarks

In order to end the COVID-19 pandemic, scientists around the world have conducted a great deal of research on SARS-CoV-2 since its initial outbreak. However, some important issues on the COVID-19 and SARS-CoV-2 are still waiting to be solved. Here, we summarized five concerned problems and discussed the possible answers based on the Three-H strategy.

Why does the pathogenicity of SARS-CoV-2 have species specificity? The interaction between HIS-SARS2 and enhancer activates expression of genes associated with inflammation and further promotes the COVID-19 progression. The sequences of HIS-SARS2 showed the higher conservation in primates (such as Rhesus and Green monkey) [129], which could explain the similar pulmonary damage to COVID-19 in rhesus macaques infected with SARS-CoV-2 [373]. Thus, the interaction between HIS-SARS2 and enhancer determines the pathogenicity of SARS-CoV-2 in specific species. Accordingly, primates could be the best choices to establish animal models of COVID-19 for mechanism research and drug development.

What is the potential mechanism of the clinical manifestations (such as cytokine storm, GGO, thrombosis, and anosmia) in COVID-19? On one hand, HIS-SARS2 activates the expression of HAS2 and induces the accumulation of hyaluronan in the SARS-CoV-2 infected host cells through targeting enhancer. As the key inflammatory mediator, hyaluronan stimulates the non-immune cells (such as fibroblasts) to release cytokines. Meanwhile, the increased hyaluronan in lung leads to the GGO in COVID-19 patients. The binding of hyaluronan to its receptor HABP2 mediates the abnormal thrombosis, which may cause the anosmia in COVID-19 [374]. On the other hand, HIS-SARS2 may promote the production of hyaluronan in distal host cells without SARS-CoV-2 infection via the transportation in exosomes. In this situation, HIS-SARS2 may activate the release of cytokines in fibroblasts through the NamiRNA-Enhancer network.

Why are the specific subpopulations (such as pregnant women, and elderly people, and male sex) susceptible to SARS-CoV-2? As one of the glycans, heparin in the surfaces of the cell facilitate the cell entry of SARS-CoV-2. Likewise, hyaluronan in the cell surfaces may stick SARS-CoV-2 and promote its infection. The pregnant women, and elderly people have higher hyaluronan level, which might enhance the invasion of SARS-CoV-2. Surprisingly, testosterone could elevate the expression of hyaluronan synthase 1 in fibroblasts [375], suggesting that the androgen in the male sex may further increase the hyaluronan production and cause the susceptibility to SARS-CoV-2.

How do we estimate the progression of COVID-19 and decrease the risk of severe COVID-19 in specific subpopulations (such as elderly people and diabetes patients)? Given that hyaluronan is closely associated with the progression of COVID-19 [281, 323], the detection of plasma hyaluronan level may be an important approach to judge the COVID-19 progression. Moreover, hyaluronan level is relatively higher in elderly people and diabetes patients. After SARS-CoV-2 infection, the complexes of hyaluronan and S protein may together aggravate the COVID-19.

Which are the novel strategies to develop the effective drugs for COVID-19 and prevent the sequelae of COVID-19? Focus on HIS and hyaluronan is one of the novel strategies for the drug development of COVID − 19. Specifically, we could design the ASO drugs targeting HIS and develop small molecular inhibitors against hyaluronan, which may block the interaction between SARS-CoV-2 and human. Similar, prevention of the hyaluronan elevation by inhibiting androgen pathway is also a strategy for the treatment of COVID-19. When it comes to the intervention therapy of COVID-19, the combination of diverse drugs (such as antiviral drugs and hymecromone) may reach a better therapeutical effect [376]. In addition, reduction of hyaluronan into the normal range might be a potential strategy to prevent the sequelae of COVID-19 though the oral administration of hyaluronan inhibitor drugs (such as hymecromone).

Taken together, Three-H is crucial for us to reconsider the underlying relationship between COVID-19 and SARS-CoV-2. The oligonucleotide drugs, inhibitors of hyaluronan synthesis, or antiallergic agents deserve our time and effort towards further validating their effectiveness in treating COVID-19 in the future.

## Data Availability

Not applicable.

## Abbreviations

ACE2: Angiotensin-converting enzyme 2
ARDS: Acute respiratory distress syndrome
ASOs: Antisense oligonucleotides
BALF: Bronchoalveolar lavage fluid
CFR: Case fatality rate
COVID-19: Coronavirus disease 2019
CRP: C-reaction protein
DPP4: Dipeptidyl peptidase 4
DMVs: Double-membrane vesicles
FDA: Food and Drug Administration
GGO: Ground-glass opacity
HIS: Human Identical Sequences
HIS-SARS2: Human Identical Sequences of SARS-CoV-2
HUVEC: Human umbilical vein endothelial cell
IP-10: IFNγ-induced protein 10
4-MU: 4-methylumbelliferone
NamiRNA: Nuclear activating miRNAs
PF4: Platelet factor 4
svRNAs: Small viral RNAs
RBD: Receptor binding domain
R0: R nought
TRS: Transcription-regulatory sequence
Three-H: HIS, hyaluronan, and hymecromone
VOC: Variants of concern
